# Effect of Vacuum Sealing Drainage on Soft Tissue Injury of Traumatic Fracture and Its Effect on Wound Recovery

**DOI:** 10.1155/2022/7107090

**Published:** 2022-09-29

**Authors:** Pengfei Nie, Canhong Zhang

**Affiliations:** ^1^Department of Orthopedics, Beilun District People's Hospital, Ningbo 315800, Zhejiang, China; ^2^Department of Orthopaedics, Quanzhou First Hospital Affiliated to Fujian Medical University, Quanzhou 362000, Fujian, China

## Abstract

**Purpose:**

The current work is mainly to explore the effect of vacuum sealing drainage (VSD) on soft tissue injury (STI) caused by traumatic fractures (TFs) and its effect on wound recovery.

**Methods:**

We first selected 90 patients with TF STI from May 2019 to May 2021, of which 40 patients (control group) received routine treatment, and the other 50 patients (observation group) were treated with VSD. The curative effect, rehabilitation (changing dressing frequency, healing time, and hospitalization time), pain severity, patient comfort, and complications were evaluated and compared.

**Results:**

The observation group exhibited a higher total effective rate, lower dressing change frequency, complication rate, and shorter healing time and hospital stay than the control group, which are statistically significant. Statistically milder pain sensation and better patient comfort were also determined in the observation group.

**Conclusions:**

VSD is effective and safe in the treatment of TF-induced sexually transmitted infections, which can effectively accelerate wound recovery while reducing pain sensation and improving patient comfort, with clinical promotion value.

## 1. Introduction

All infections and injuries of soft tissues are no small challenge for patients and medical staff. Soft-tissue disorders are essentially a manifestation of systemic health that requires proper diagnosis, resuscitation, management, and support [1]. Traumatic fractures (TFs), often accompanied by open injuries, are prone to occur in all kinds of soft tissues in various sports [2–4]. These soft tissue injuries (STIs) can easily lead to the reduction of soft tissue coverage and the destruction of local blood supply that adversely affect fracture healing, resulting in some patients experiencing wound infection and necrosis due to wound contamination [5]. From an ergonomic point of view, the mechanical behavior of the whole tissue will change dramatically if the soft tissue is damaged, which in turn suggests that biomechanical changes caused by STIs can be used to detect the severity of the injury [6, 7]. In any event, all kinds of STIs must be considered when detecting and treating TFs [8]. Hence, research on the treatment of STIs and wound recovery has important practical value for improving patient outcomes and avoiding wound infection and necrosis.

Vacuum sealing drainage (VSD) has a wide range of applications in wound care as well as clinical treatment at present [9]. In the management of various diseases, including open abdominal wounds, burns, pressure sores, sternal wounds, and obese patients after bariatric surgery, VSD is often used [10, 11]. This technique plays an important role in preventing wound infections of closed incisions as well as surgical site complications [12]. The treatment begins with the construction of a closed, sealing system that applies negative pressure (suction) to the wound surface. The wound is then covered or wrapped with an open-cell foam or gauze dressing and sealed with a sealing cover. And subsequently, intermittent or continuous suction is maintained by connecting a suction pipe from the wound dressing to a vacuum pump and a waste liquid collector [13]. In this paper, we will explore the curative effect of VSD in treating STIs resulted from TFs and its impact on wound recovery.

## 2. Methods

### 2.1. General Information

We selected 90 patients with STIs attributed to TFs admitted from May 2019 to May 2021, 40 of whom received routine treatment and were included in the control group, and the other 50 cases were additionally treated with VSD and used as the observation group. The control and observation groups were not statistically different in general data (*P* > 0.05).

Inclusion criteria were as follows: (1) diagnosis of TFs by the presence of clinical presentations, medical history, computerized tomography (CT) or ultrasound examination, and surgical examination [14]; (2) Gustilo classification type II or III [15]; (3) mentally normal with no history of mental illness; (4) ability to clearly express the discomfort, without communication barrier. Exclusion criteria were as follows: (1) serious primary diseases of heart, liver, kidney, and other VITAL organs; (2) serious diseases of the blood system; (3) history of anti-infection, immunosuppressants, hormones, and other drugs within one month before treatment.

Informed consent was provided by patients or their families, and the study was conducted after obtaining approval from the Hospital Ethics Committee.

### 2.2. Therapies

The control group received routine treatment. In the emergency operating room, the wound was precleaned with clean water and the necrotic tissue was removed, followed by disinfection with iodophor and hydrogen peroxide. In the case of wound degloving, a sharp knife was used to puncture the mesh for cleaning and disinfection; any foreign bodies in the wound were removed before wound cleaning and disinfection. After debridement, one-stage fracture repair was performed, and an appropriate amount of wound secretions were collected for bacterial culture. Drug sensitivity tests were performed on infected patients, and anti-infection dressings were selected based on the results of bacteria and drug sensitivity culture. Then, appropriate saline and antibiotics mixed with wet gauze were cut, gently applied to the wound surface, and wrapped up with cotton bandages. Dressing change was conducted 1 day later. Thereafter, the dressing was changed every 1–3 days according to the healing condition of the wound until the wound could be closed by second-stage surgery (suturing or skin grafting, skin flap repair, etc.). On this basis, the observation group was treated with VSD. The preoperative treatment, operation, bacterial culture, and drug sensitivity test of patients with primary fracture repair were the same as those of the control group, followed by 75% ethanol disinfection. The VSD dressing was cut according to the size and shape of the wound, and the translucent membrane was pasted to seal the wound (3 cm longer than the VSD dressing). Then the two drainage tubes were connected, one of which maintained VSD negative pressure at 26.6–59.8 kPa, and the other was continuously irrigated with anti-infective drugs +500 mL normal saline according to the results of bacteria and drug sensitivity culture. The dressing was changed once every 5–7 days in patients with turbid drainage fluid and once every 7–10 days in those with clear drainage fluid. The duration of VSD was determined according to the patient's wound size and the infection situation until the second-stage operation can be performed to close the wound (suture or skin grafting, skin flap repair, etc.)

### 2.3. Measurement Indicators

#### 2.3.1. Rehabilitation

The rehabilitation of the two cohorts of patients, evaluated from dressing change frequency, healing time, and length of hospital stay (LOS), was compared.

#### 2.3.2. Pain Severity and Comfort Status

The pain severity and patent comfort, assessed by the Visual Analog Scale (VAS) and Kolcaba's General Comfort Questionnaire (GCQ) [16, 17], respectively, were counted in both cohorts.

#### 2.3.3. Total Efficiency

The total effective rate was also statistically compared. Evaluation criteria [4]were as follows: cured: the wound healed and the epidermis survived well without the need for dressing changes; effective: obviously reduced wound area, with partially survived skin flap, reduced secretions, but the need for dressing changes; ineffective: little relief of symptoms and signs before and after treatment, with no obvious change in wound surface and secretions. The total effective rate = (cure + effective) cases/the total number of cases in this group × 100%.

#### 2.3.4. Complication Rate

The incidence of complications (infection, skin necrosis, amyotrophy, and osteomyelitis) was statistically compared.

### 2.4. Statistics and Methods

SPSS22.0 (Asia Analytics Formerly SPSS China) was employed for synthetic data analysis. Categorical variables (*n*(%)) were tested using the *χ*^2^ test, and continuous variables (*X* ± *S*) were compared by the *t*-test before and after surgery within the group. *P* < 0.05 was the significance threshold.

## 3. Results

### 3.1. General Data

As shown in Table 1, the two cohorts were comparable in gender, age, body mass index (BMI), and other general data (*P* > 0.05).

### 3.2. Rehabilitation

**Figure 1:**
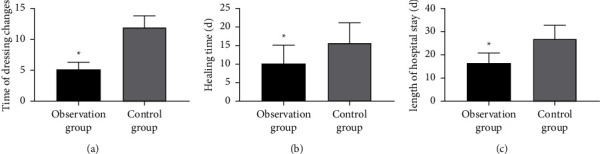
Comparison of rehabilitation: (a) times of dressing changes: the observation group had fewer times of dressing changes than the control group (*P* < 0.05); (b) healing time: the observation group had a shorter healing time than the control group (*P* < 0.05); (c) length of hospital stay: the observation group had less length of hospital stay than the control group (*P* < 0.05). Note ^*∗*^ means that compared with the control group, *P* < 0.05.

Comparing patients' postoperative rehabilitation (Figure 1), we can see that the dressing change frequency, healing time, and LOS were significantly less in the observation group than in the control group (*P* < 0.05).

### 3.3. Pain Severity and Comfort Status

**Figure 2:**
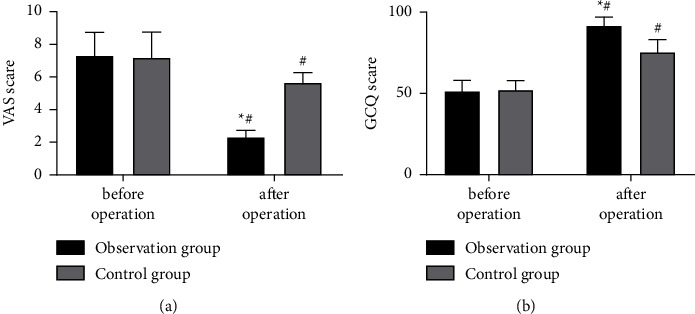
Comparison of rehabilitation: (a) VAS score: reduced postoperative VAS scores were observed in both cohorts, with a lower score in the observation group versus the control group (*P* < 0.05); (b) GCQ score: elevated postoperative GCQ scores were determined in both cohorts, with a more significant increase in the observation group compared with the control group (*P* < 0.05). Note ^*∗*^ means *P* < 0.05 compared with the control group; ^#^ means *P* < 0.05 compared with the postoperative score within the group.

The intergroup comparison (Figure 2) of postoperative pain severity and comfort status revealed obviously alleviated pain and improved patient comfort in both cohorts. And in comparison with controls, the observation group had a lower VAS score and a higher GCQ score (*P* < 0.05).

### 3.4. Total Effective Rate

Statistics on overall treatment efficacy (Table 2) revealed an evidently higher total effective rate in the observation group (*P* < 0.001).

### 3.5. Complication Rate

The statistics on postoperative complications (Table 3) identified a higher complication rate in the control group as compared to the observation group (*P* < 0.05).

## 4. Discussion and Conclusion

Fractures that occur after high-energy trauma are usually accompanied by various intra-articular lesions such as ligamentum teres injury, loose body, cartilage injury, and trauma of soft tissue like the lip, which can cause ischemic necrosis, post-traumatic osteoarthritis, and even long-term disability in severe cases [18–20]. Therefore, for STIs of TFs, it is necessary to use the correct treatment. In this section, we will study the effectiveness of VSD on TF-induced STIs through various indicators.

This study found significant changes in the postoperative dressing change frequency, healing time, and LOS in both cohorts, with fewer times of dressing changes, and shorter healing time and LOS in the observation group using VSD. VSD has been proven to be effective in treating chronic and complex wounds, with the wound dehiscence rate approximately halved with this technology [21]. VSD renders benefits to various types of surgeries. In breast surgery with easy recurrence and multiple complications, for example, the use of VSD can reduce seroma and its sequelae [22, 23]. As an auxiliary treatment method for open wounds, VSD will apply controllable negative pressure to the wounds through various equipment and professional dressings, and transfer wound liquid to appropriate containers, which can create a wound environment conducive to healing by removing infected materials and exudates, reducing edema, and promoting perfusion and granulation [24, 25]. Therefore, compared with the control group which only used conventional treatment, the wound healing of the observation group was far better with a higher total effective rate because of the use of VSD. Similarly, the complication rate in the observation group was not as high as that in the control group due to the effect of VSD in reducing inflammation and infection. From the above, it is clear that VSD, as an auxiliary treatment method, can greatly improve the wound healing effect and reduce the occurrence of various complications in patients. This has been confirmed in other researchers' analyses. For example, in the study of Cai et al. [26], VSD validly promoted postoperative wound healing in patients with closed calcaneal fractures, shortened the wound healing time, and lowered the incidence of wound complications, which is consistent with our findings. And as reported by Zhang et al. [27], compared with conventional dressing change intervention, orthopedic trauma patients treated with VSD had a higher total effective rate, lower dressing change frequency, and shorter wound healing time with certain security, which contributed to effectively reduced risk of adverse events such as postoperative infections and lower extremity deep vein thrombosis, similar to the results of our study.

This study also found evidently changed VAS and GCQ scores in both cohorts, with a lower VAS score and a higher GCQ score in the observation group. Consistently, Jiao et al. [28] reported significantly reduced pain severity during dressing change by VSD technique in patients with sural neurocutaneous flap transplantation in the foot and ankle. This technique has also been applied to examine patients with nonspecific hip, knee, and low back pain. Studies have found that associated soft tissues are affected following similar fractures and a range of similar orthopedic diseases [29]. This type of soft tissue defect of the skin in various parts of the body caused by external trauma is very common in clinical treatment [30]. Since some parts of the body (forearm, hand, knee joint, skin, and soft tissue in front of tibia and foot) have thinner skin texture, less subcutaneous tissue, and are located in the exposed parts of the limbs, ineffective repair of any defect will predispose patients to long-lasting pain and abnormal discomfort, which will exert a great impact on patient's mobility and life quality [31–33]. Combined with the results of this study, we can draw a conclusion that, for patients in the observation group who used VSD, there were faster recoveries and better curative effects, contributing to less pain and discomfort compared with the control group.

This study is unique in that it comprehensively analyzes the clinical efficacy and safety of the VSD technique from multiple aspects such as efficacy, rehabilitation, pain degree, patient comfort, and complications, and confirms its effectiveness in the treatment of patients with TFs and STIs, which provides a new choice for the management of such patients. But it still has room for improvement. This time, we failed to explore patients' postoperative life quality, nor have we investigated their degree of cooperation during the operation and the improvement of psychological state. These survey indicators will be taken into account in future studies to continuously improve the clinical treatment plan.

Taken together, VSD is effective in treating TF-induced STIs, contributing to effectively accelerated wound recovery while ensuring patient safety, which deserves clinical popularization.

## Figures and Tables

**Table 1:** General data.

Classification	Observation group (*n* = 50)	Control group (*n* = 40)	*t*/*χ*^2^	*P*
Sex			0.02	0.887
Male	27 (54.00)	21 (52.50)		
Female	23 (46.00)	19 (47.50)		

Age (years old)	40.12 ± 5.75	39.08 ± 6.85	0.78	0.436

BMI (kg/m^2^)	20.62 ± 3.26	20.42 ± 4.19	0.25	0.800

Gustilo classification			0.24	0.627
II	30 (60.00)	26 (65.00)		
III	20 (40.00)	14 (35.00)		

Site of injury			0.03	0.985
Upper limbs	12 (24.00)	9 (22.50)		
Thigh	28 (56.00)	23 (57.50)		
Lower leg	10 (20.00)	8 (20.00)		

Smoking			0.38	0.538
Yes	40 (80.00)	34 (85.00)		
No	10 (20.00)	6 (15.00)		

Drinking			0.15	0.701
Yes	37 (74.00)	31 (77.50)		
No	13 (26.00)	9 (22.50)		

**Table 2:** Total effective rate of two groups of patients.

Classification	Observation group (*n* = 50)	Control group (*n* = 40)	*χ* ^2^	*P*
Cured	25 (50.00)	12 (30.00)	—	—
Effective	23 (46.00)	16 (40.00)	—	—
Ineffective	2 (4.00)	12 (30.00)	—	—
Total effective rate	48 (96.00)	28 (70.00)	11.44	<0.001

**Table 3:** Complications in two groups.

Classification	Observation group (*n* = 50)	Control group (*n* = 40)	*χ* ^2^	*P*
Infection	2 (4.00)	4 (10.00)	—	—
Skin necrosis	1 (2.00)	4 (10.00)	—	—
Amyotrophy	0 (0.00)	2 (5.00)	—	—
Osteomyelitis	0 (0.00)	4 (10.00)	—	—
Incidence of complications (%)	3 (6.00)	14 (35.00)	12.20	<0.001

## Data Availability

The labeled dataset used to support the findings of this study is available from the corresponding author upon request.
